# A Molecular and Functional Investigation of the Anabolic Effect of an Essential Amino Acids’ Blend Which Is Active In Vitro in Supporting Muscle Function

**DOI:** 10.3390/nu18020323

**Published:** 2026-01-20

**Authors:** Lorenza d’Adduzio, Melissa Fanzaga, Maria Silvia Musco, Marta Sindaco, Paolo D’Incecco, Giovanna Boschin, Carlotta Bollati, Carmen Lammi

**Affiliations:** 1 Department of Pharmaceutical Sciences, University of Milan, Via Mangiagalli, 25, 20133 Milan, Italy; 2 Department of Food, Nutrition and Environmental Sciences, University of Milan, Via Celoria, 2, 20133 Milan, Italy

**Keywords:** essential amino acids’ blend, Caco-2 cells, GLUT-4, myotubes

## Abstract

**Background/Objectives**: Essential amino acids’ (EAAs) biological effects depend on both gastrointestinal stability and intestinal bioavailability. A commercially available EAA blend has previously shown to be highly bioaccessible and able to inhibit the DPP-IV enzyme both directly and at a cellular level following simulated digestion in vitro. In light with this consideration, the present study aimed to evaluate the intestinal in vitro bioavailability of GAF subjected to INFOGEST digestion (iGAF) and to investigate the metabolic effects of its bioavailable fraction on muscle cells using an integrated Caco-2/C2C12 co-culture model. **Methods**: Differentiated Caco-2 cell lines were treated with iGAF, and amino acid transport was quantified by ion-exchange chromatography. The basolateral fraction containing bioavailable EAAs was used to treat differentiated C2C12 myotubes for 24 h. Western blot analyses were performed to assess the activation of anabolic and metabolic pathways, including mTOR, Akt, GSK3, AMPK and GLUT-4. **Results**: More than 50% of each EAA present in iGAF crossed the Caco-2 monolayer, with BCAAs and phenylalanine particularly enriched in the basolateral fraction. Exposure of C2C12 myotubes to the bioavailable iGAF stimulated mTORC1 activation and increased the phosphorylation of Akt and GSK3, indicating an enhanced anabolic response. At a cellular level, iGAF also elevated the p-AMPK/AMPK ratio, suggesting activation of energy-sensing pathways. Moreover, GLUT4 protein levels and glucose uptake were significantly increased. **Conclusions**: The study focuses exclusively on a cellular model, and results suggested that iGAF is highly bioavailable in vitro and that its absorbed fraction activates key anabolic and metabolic pathways of skeletal muscle cells, enhancing both protein synthesis signaling and glucose utilization in vitro.

## 1. Introduction

Amino acids are the building blocks of proteins and act as substrates for protein synthesis [1]. Isoleucine, leucine, lysine, methionine, phenylalanine, threonine, tryptophan and valine are considered essential amino acids (EAAs) as they cannot be produced de novo, or because endogenous synthesis is insufficient to satisfy physiological requirements, making their dietary intake necessary, primarily from protein-rich sources such as meat, dairy, legumes and certain grains [2]. It is known that EAAs play a critical role in maintaining cellular homeostasis, protein synthesis and overall metabolic balance in humans and other higher organisms [1,2].

In addition to providing the building blocks for structural and enzymatic proteins, EAAs also act as precursors for key biomolecules involved in neurotransmission (e.g., serotonin from tryptophan, dopamine from phenylalanine and tyrosine), methyl-group transfer (through methionine-derived S-adenosylmethionine) and redox regulation (such as cysteine synthesis via methionine metabolism) [3]. In recent years, the demand of EAA supplementation has seen rapid growth, especially in promoting physical performance in athletes [4] and decreasing muscle atrophy correlated with aging and sarcopenia [5,6]. From a physiological standpoint, EAAs are essential for muscle protein synthesis, tissue repair, immune competence and hormonal regulation [7]. Notably, the branched-chain amino acids (BCAAs) isoleucine, leucine and valine have been shown to play a pivotal role in modulating mTOR signaling pathways, which govern muscle hypertrophy and metabolic adaptation [8]. Furthermore, adequate EAA intake is closely linked to nitrogen balance, metabolic health, and cognitive performance, while their deficiency can lead to growth retardation, immune dysfunction, and impaired recovery from injury or stress [9]. EAAs, particularly BCAAs, exert a dual role in cellular physiology by serving both as substrates for protein synthesis and as potent metabolic regulators that modulate glucose uptake and energy substrate utilization, influencing several key anabolic and metabolic pathways [10,11]. More specifically, at the molecular level, EAAs activate nutrient-sensitive signaling cascades that coordinate cellular growth and metabolism. Among them, the mechanistic target of the rapamycin complex 1 (mTORC1) pathway represents the principal hub integrating amino acid availability, energy status and hormonal cues. Leucine, in particular, is one of the most potent activators of mTORC1, primarily through its interaction with the Rag GTPases and the lysosomal localization of mTOR, which leads to the phosphorylation of its downstream effectors, ribosomal protein S6 kinase 1 (S6K1) and eukaryotic translation initiation factor 4E-binding protein 1 (4E-BP1), thereby stimulating mRNA translation, ribosome biogenesis and global protein synthesis [12]. This mechanism underlies the strong anabolic effect of EAAs, especially in skeletal muscle tissue, where increased amino acid availability directly enhances the rate of myofibrillar protein accretion. Moreover, EAAs play a crucial role in the regulation of glucose metabolism. They stimulate insulin secretion from pancreatic β-cells and further augment insulin signaling in peripheral tissues, including skeletal muscle and adipose tissue. Through the activation of the PI3K-Akt pathway, EAAs enhance the translocation of GLUT4 glucose transporters to the plasma membrane, thereby increasing glucose uptake. This coordinated action ensures that sufficient energy and carbon skeletons are available to sustain the energetically demanding process of protein synthesis. Moreover, insulin and EAAs act synergistically to suppress proteolysis, promoting a positive net protein balance within the cell. In this context, a commercially available EAA blend, known as Gunaminoformula (GAF), shows a defined qualitative and quantitative ratio of EAAs (L-leucine, 200 mg/g; L-valine, 160 mg/g; L-isoleucine, 150 mg/g; L-lysine, 140 mg/g; L-phenylalanine, 130 mg/g; L-threonine, 110 mg/g; L-methionine, 70 mg/g; L-tryptophan, 40 mg/g, Table A1), specifically designed to support EAA requirements in various populations—such as athletes, including providing support in muscle recovery and performance—and prevent age-related muscle loss as well as maintain sufficient amino acid intake in individuals on restrictive diets, such as vegetarians or vegans. Recent evidence demonstrated that GAF’s amino acids’ composition is highly stable and bioaccessible after INFOGEST-simulated gastrointestinal digestion [13]. In order to characterize the effect of the EAA blend on in vitro muscle cells, a developed co-culture system, employing both intestinal differentiated Caco-2 and C2C12 myotubes cells, was used to dynamically study the anabolic effect of the bioaccessible and bioavailable iGAF on the energy and glucose metabolism of differentiated C2C12 cells. In this model, Caco-2 cells were cultured on the apical compartment of a Transwell system, while differentiated C2C12 myotubes were cultured in the basolateral compartment. This configuration allows metabolites absorbed by the intestinal cells to reach muscle cells, making it possible to evaluate their impact on energy and glucose metabolism (measuring the activation of Akt-mTORC1-GSK3 and AMPK pathways, respectively) directly in C2C12 myotubes cultured on the bottom side of a Transwell system.

## 2. Materials and Methods

### 2.1. Chemicals

The Gunaminoformula sample was provided by Guna S.p.A. Dulbecco’s Modified Eagle’s Medium (DMEM), L-glutamine, fetal bovine serum (FBS), phosphate-buffered saline (PBS), penicillin–streptomycin, and 96- and 12-well plates were purchased from Euroclone (Milan, Italy). Sigma-Aldrich (St. Louis, MO, USA) supplied morpholinoethane sulfonic acid (MES), 4-(2-hydroxyethyl)-1-piperazineethanesulfonic acid (HEPES), hydrochloric acid (HCl), bovine serum albumin (BSA), RIPA buffer and the antibody against β-actin. Horse serum came from Sigma-Aldrich (St. Louis, MO, USA), Caco-2 cells from INSERM (Paris, France), C2C12 cells from ATCC (HB-8065, ATCC from LGC Standards, Milan, Italy), and 12-MW black plates from Biosigma S.p.A.—Cona (VE), Italy. The antibodies mTOR (66888-1-Ig), AMPKα (10929-2-AP), AKT (10176-2-AP), GSK3β (22104-1-AP) were from Proteintech (Rosemont, IL, USA). p-AMPKα (2535S), p-AKT (4060S), p-GSK3α/β (9331S), GLUT-4 (2213S), and p-mTOR (2971S) were purchased from Cell Signaling Technology (Danvers, MA, USA). The antibody anti-β-actin (Sigma, A5441) was from Sigma-Aldrich (St. Louis, MO, USA). Na-orthovanadate inhibitors and goat anti-rabbit Ig-HRP were purchased from Mini protean TGX pre-cast gel 7.5%, and Mini nitrocellulose Transfer Packs were purchased from BioRad (Hercules, CA, USA). The microscope used was Axio Vert.A1 TL/RL-LED s/n 3849000804 (ZEISS, Hombrechtikon, Switzerland), and the software used for acquiring cell pictures was ZEN Microscope Software (Version 3.6).

### 2.2. Cell Culture

According to a previously optimized protocol, Caco-2 and C2C12 cells were routinely maintained in DMEM supplemented with 25 mM glucose, 3.7 g/L NaHCO_3_, 4 mM stable L-glutamine, 1% non-essential amino acids, 100 U/L penicillin, 100 μg/L streptomycin (complete medium), and 10% heat-inactivated FBS. Cultures were incubated at 37 °C in a humidified atmosphere containing 90% air and 10% CO_2_.

### 2.3. Caco-2 and C2C12 Cell Differentiation

For Caco-2 cell differentiation, cells were seeded on polycarbonate Transwell inserts (12 mm diameter, 0.4 μm pore size; Corning Inc., Lowell, MA, USA) at a density of 3.5 × 10^5^ cells/cm^2^ in complete medium containing 10% fetal bovine serum (FBS) added to both the apical (AP) and basolateral (BL) compartments. After 2 days, once a confluent monolayer had formed, the medium in both compartments was replaced with FBS-free medium beginning on day 3 post-seeding. Cells were maintained under these conditions for 18–21 days, with medium changes performed three times per week [14]. For C2C12 differentiation, two days after seeding the growth medium was replaced with differentiation medium consisting of DMEM supplemented with 25 mM glucose, 3.7 g/L NaHCO_3_, 4 mM stable L-glutamine, 1% non-essential amino acids, 100 U/L penicillin and 100 μg/L streptomycin (complete medium), with an additional 2% heat-inactivated horse serum.

### 2.4. 3-(4,5-Dimethylthiazol-2-yl)-2,5-Diphenyltetrazolium Bromide (MTT) Assay

A total of 2 × 10^4^ C2C12 cells/well were seeded in 96-well plates. To evaluate the impact of iGAF on cells’ viability, C2C12 cells were treated with iGAF 10 mg/cm^2^ or vehicle (H_2_O) in complete growth medium for 2 h, at 37 °C under a 5% CO_2_ atmosphere, following the procedure previously reported [15].

### 2.5. Evaluation of Caco-2 Monolayer Integrity

The transepithelial electrical resistance (TEER) of differentiated Caco-2 monolayers was assessed at 37 °C using a Millicell voltmeter (Millipore Co., Billerica, MA, USA). Measurements were taken immediately before treatment and after 15, 30, 60, and 120 min of incubation. Only monolayers displaying TEER values comparable to those of untreated control cells were included in the study.

### 2.6. Amino Acid Uptake by Intestinal Monolayers

iGAF intestinal absorption was evaluated using a transport buffer consisting of 137 mM NaCl, 5.36 mM KCl, 1.26 mM CaCl_2_, 1.1 mM MgCl_2_, and 5.5 mM glucose. To mimic physiological small intestinal conditions, apical solutions were adjusted to pH 6.0 with 10 mM morpholinoethanesulfonic acid, while basolateral (BL) solutions were maintained at pH 7.4 using 10 mM N-2-hydroxyethylpiperazine-N′-4-butanesulfonic acid. Before the transport assay, cells were equilibrated in HBSS for 15 min at 37 °C. We previously defined that cell treatment conditions were designed to mimic the physiological intestinal absorption of the digested iGAF product. Based on the estimated duodenal absorptive surface (≈2450 cm^2^) [16], the in vitro dose was scaled to a fixed surface load of 10 mg/cm^2^ (referring to well area). Each well received an amount of iGAF proportional to its surface area. Under these conditions, MTT results previously showed that at 10 mg/cm^2^ iGAF did not cause negative effects on Caco-2 cells’ viability [13]. Accordingly, iGAF 10.7 mg/well (equivalent to 10 mg/cm^2^) was dissolved in 500 μL of apical transport buffer and added to the apical chamber (having an area of 1.12 cm^2^), whereas 700 μL of BL transport buffer was added to the BL chamber. After 1 h of incubation at 37 °C, samples from both the apical and BL compartments were collected and analyzed by ion-exchange chromatography using a Biochrom 30+ amino acid analyzer (Erreci, Milan, Italy).

### 2.7. Amino Acid Analysis

The iGAF and BL solutions were diluted 1:4 in 0.2 N lithium citrate buffer (pH 2.2), filtered through a 0.2 μm membrane (Millipore, Milford, MA, USA), and analyzed by ion-exchange chromatography using a Biochrom 30+ amino acid analyzer (Erreci, Milan, Italy), according to the method reported by Hogenboom et al. [17]. Individual amino acids were quantified using five-point calibration curves.

### 2.8. Co-Culture Caco-2/C2C12 Cell System Development

The biological effects of adsorbed and bioavailable iGAF on differentiated C2C12 cells was assessed by developing a co-culture system of Caco-2 and C2C12 cells to investigate the impact of amino acids absorbed at the intestinal level on myotubes. Specifically, Caco-2 cells (3.5 × 10^4^ cells/well) were seeded onto Transwell filters and differentiated over 21 days. In parallel, C2C12 cells (2 × 10^5^ cells/well) were seeded on 12-well plates and differentiated into myotubes, expressing characteristic muscle proteins. Caco-2 differentiation was monitored by measuring transepithelial electrical resistance (TEER), while C2C12 differentiation was assessed by observing myotube formation. When both the cell lines were differentiated, with polarized Caco-2 cells that formed a monolayer mimicking the absorbent intestinal barrier formation and C2C12 that progressively changed their shape, becoming elongated and developing structures involved in contraction [18], the Transwell system with differentiated Caco-2 cells was moved on the differentiated C2C12 cells plate. Caco-2 cell experiments were set up to simulate the physiological conditions of intestinal iGAF absorption. Therefore, we considered the previously defined cellular treatments condition, and Caco-2 cells were treated in the apical (AP) compartment with iGAF 10.7 mg/well for 2 h [13]. TEER values were monitored throughout the 2 h transport experiment. At the endpoint, the basolateral (BL) solution containing absorbed iGAF was applied to differentiated Caco-2 cells for 24 h. The following day, the cells were lysed and used for Western blot analysis.

### 2.9. Western Blot Analysis

Following 24 h of exposure to the basolateral (BL) solution containing bioavailable iGAF, C2C12 cells were scraped into 40 μL of ice-cold lysis buffer (RIPA supplemented with a protease inhibitor cocktail, 1:100 PMSF, and 1:100 sodium orthovanadate) and transferred to pre-chilled microcentrifuge tubes. The lysates were centrifuged at 16,060× *g* for 15 min at 4 °C, and the supernatants were collected into fresh, chilled tubes. Total protein concentrations were determined using the Bradford assay, after which 50 μg of protein per sample was resolved on a pre-cast 7.5% SDS-PAGE gel at 130 V for 45 min. Proteins were subsequently transferred onto nitrocellulose membranes (Mini Nitrocellulose Transfer Packs) using the Trans-Blot Turbo system set to 1.3 A and 25 V for 7 min. Following blocking with milk, membranes were incubated with primary antibodies directed against AMPK, p-AMPKα (Thr172), Akt, p-Akt (Ser473), GSK3, p-GSK3, GLUT-4, mTOR, p-mTOR, and β-actin. Signal detection was achieved using HRP-conjugated secondary antibodies and a chemiluminescent substrate, and band intensities were quantified using Image Lab Software, version 3.01 (Bio-Rad, Hercules, CA, USA). β-Actin served as the internal loading control for normalization.

### 2.10. Fluorescent Glucose Uptake Cell-Based Assay

C2C12 cells (4 × 10^4^ cells/well) were seeded in 12-well black plates and maintained in complete growth medium. Upon reaching confluence, the medium was replaced with differentiation medium containing 2% horse serum until differentiation was observed. Differentiated Caco-2 cells on Transwell inserts were then placed above the differentiated C2C12 cells (with their medium switched to low-glucose complete DMEM) and treated with iGAF 10.7 mg/well in low-glucose complete DMEM for 2 h. Following this incubation, the basolateral (BL) solution, containing absorbed and bioavailable iGAF, was applied to C2C12 cells for 24 h. The next day, the BL solution was removed, and 150 μL/well of 2-NBDG (100 μg/mL) in serum-free low-glucose DMEM was added for 10 min at 37 °C. Excess 2-NBDG was aspirated without disturbing the cell layer, and cells were washed twice with 100 μL of the cell-based assay buffer. Glucose uptake was then measured and quantified using a Synergy H1 fluorescent plate reader (BioTek) with excitation and emission wavelengths of 485 and 535 nm, respectively.

### 2.11. Statical Analysis

**Figure 1:**
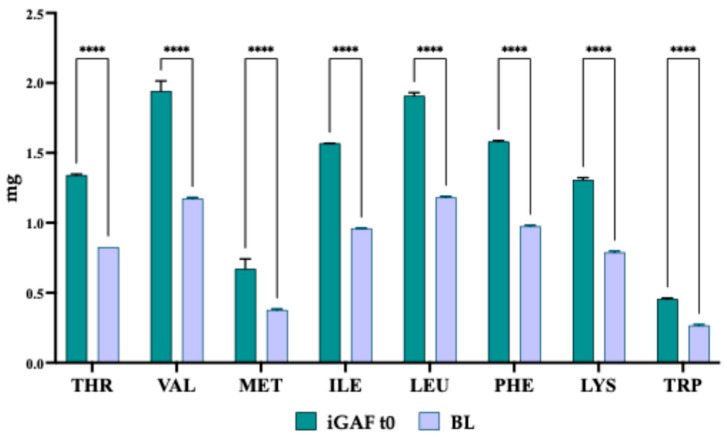
Amino acid quantification in iGAF sample (iGAF t0) and in Transwell system’s basolateral solution (BL). Green lines refer to the a.a. content in the bioaccessible iGAF sample (iGAF t0); lilac lines refer to the a.a. present in the BL solution. The data points represent the averages ± SD of 3 independent experiments (technical replicates) performed in triplicate (biological replicates). All data sets were analyzed by two-way ANOVA followed by Tukey’s post hoc test. (****) *p* < 0.0001. THR: threonine; VAL: valine; MET: methionine; ILE: isoleucine; LEU: leucine; PHE: phenylalanine; LYS: lysine; TRP: tryptophan. iGAF t0: GAF sample subjected to INFOGEST protocol; BL: basolateral solution of the Transwell system.

**Figure 2:**
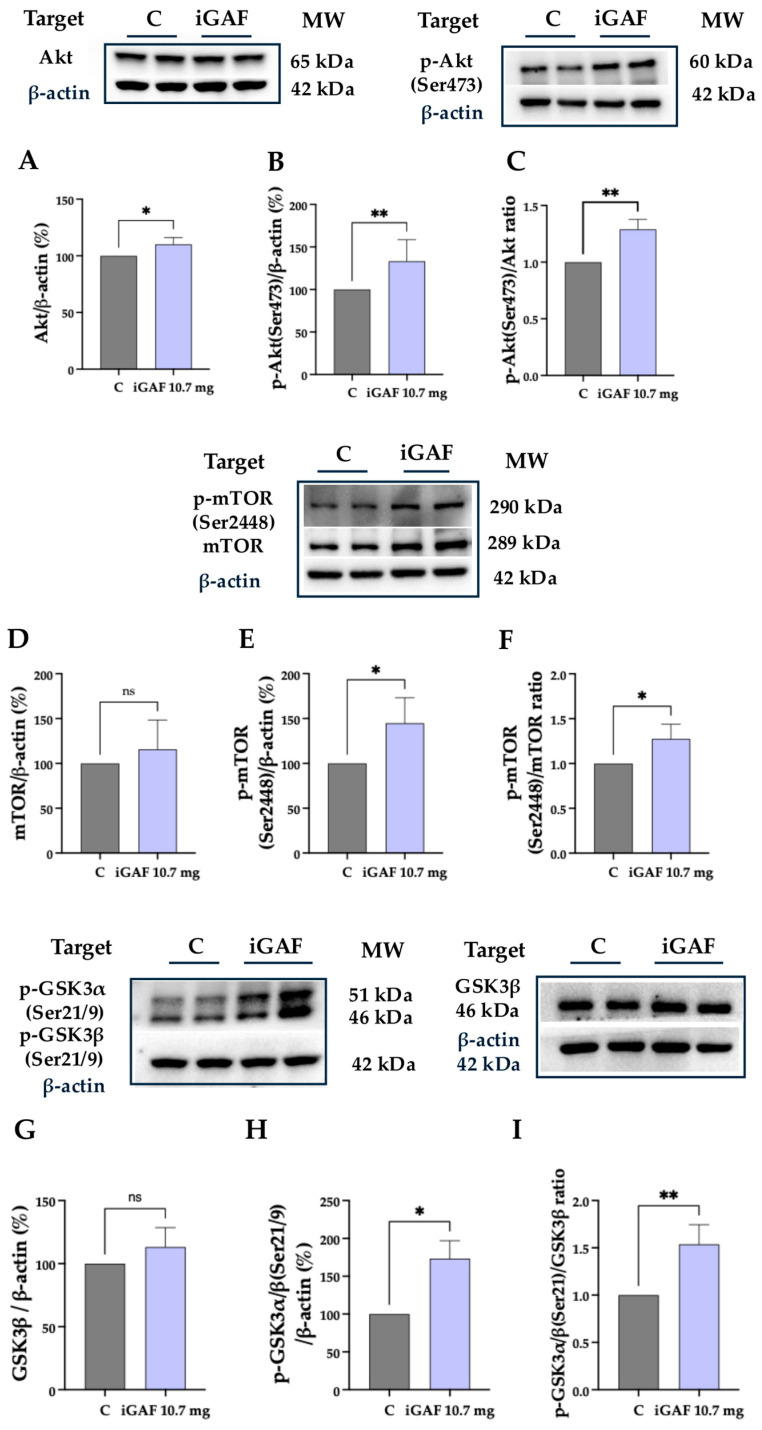
Effect of bioavailable iGAF on Akt-mTORC1/GSK3 pathway targets. (**A**) Akt protein levels, (**B**) p-AKT protein levels, (**C**) p-Akt/AKT ratio, (**D**) mTOR protein levels, (**E**) p-mTOR (Ser2448) protein levels, (**F**) p-mTOR (Ser2448)/mTOR ratio, (**G**) GSK3β, (**H**) p-GSK3α/β (Ser21/9) protein levels, and (**I**) p-GSK3α/β (Ser21/9)/GSK3β ratio. Gray lines indicate the control (untreated) cells; lilac lines indicate the treated condition with iGAF 10.7 mg/well. The data points represent the averages ± SD of at least 3 independent and parallel experiments, with each biological replicate loaded in duplicate. All the data sets were analyzed with a *t*-test. C: control, untreated cells. ns: not significant. (*) *p* < 0.05; (**) *p* < 0.01. Western blot images were obtained from membranes that were cut and incubated separately with the corresponding primary antibodies in order to reduce costs and experimental time.

**Figure 3:**
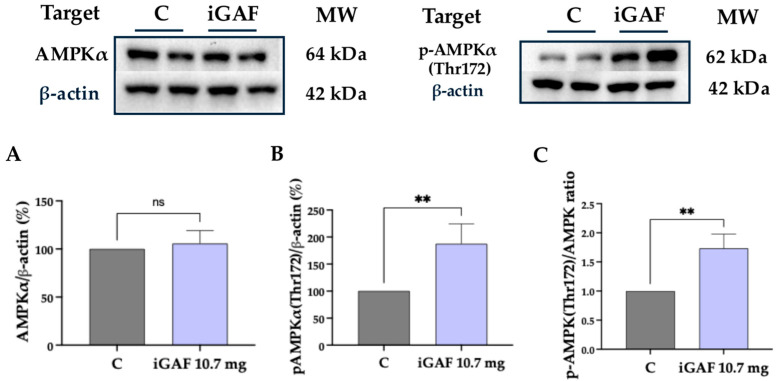
Effect of bioavailable iGAF on molecular targets involved in C2C12 cells’ energy metabolism. (**A**) AMPKα and (**B**) p-AMPKα (Thr172) protein levels, (**C**) p-AMPKα (Thr172)/AMPK ratio. Grey lines refer to untreated cells; lilac lines refer to cells treated with iGAF 10.7 mg. The data points represent the averages ± SD of at least 3 independent and parallel experiments, with each biological replicate loaded in duplicate. All the data sets were analyzed with a *t*-test. C: control. ns: not significant. (**) *p* < 0.01. Western blot images were obtained from membranes that were cut and incubated separately with the corresponding primary antibodies in order to reduce costs and experimental time.

**Figure 4:**
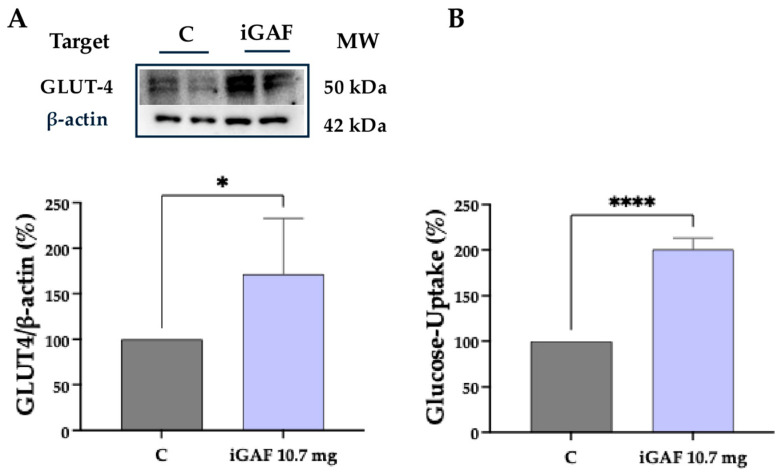
Effect of bioavailable iGAF on glucose metabolism in C2C12 cells. (**A**) Bioavailable iGAF’s impact on GLUT-4 protein levels. (**B**) Quantitative analysis of glucose uptake in C2C12 cells. Grey lines refer to untreated cells; lilac lines refer to cells treated with iGAF 10.7 mg/well. The data points represent the averages ± SD of at least 3 independent and parallel experiments, with each biological replicate loaded in duplicate. All the data sets were analyzed with a *t*-test. (*) *p* < 0.05. (****) *p* < 0.0001. C: control; untreated cells. Western blot images were obtained from membranes that were cut and incubated separately with the corresponding primary antibodies in order to reduce costs and experimental time.

Results were presented as the mean ± standard deviation (s.d.), and all measurements were carried out at least in triplicate. All the data sets were checked for normal distribution by D’Agostino and using Pearson’s test. Since they are all normally disturbed with *p*-values < 0.05, we proceeded with statistical analyses with *p*-values < 0.05 deemed significant. In particular, the data sets reported in Figure 1 were analyzed by two-way ANOVA followed by Tukey’s post hoc test, while the data sets reported in Figure 2, Figure 3, Figure 4 and Figure S2 were analyzed using a *t*-test (Graphpad Prism 9, GraphPad Software, La Jolla, CA, USA).

## 3. Results

### 3.1. Assessment of Bioaccessible iGAF Amino Acids’ Trans-Epithelial Trasport by Differentiated Human Intestinal Caco-2 Cells

GAF’s amino acid composition analysis previously demonstrated good EAA bioaccessibility, with all EAAs being stable during in vitro INFOGEST digestion [13]. To investigate iGAF’s bioavailability at the intestinal level, we employed differentiated Caco-2 cells as an in vitro model of the intestinal barrier [19,20,21]. Differentiated Caco-2 cells were treated with iGAF 10 mg/cm^2^ (10.7 mg/well). According to TEER values (Figure S1), iGAF did not alter the Caco-2 cells’ monolayer integrity, suggesting that the tightness of the cell monolayer was maintained after iGAF treatment. Thus, after 2 h of incubation, a BL solution was collected and analyzed. The results (Figure 1) demonstrate that more than 50% of each amino acid (61.46% Thr, 61.74% Val, 61.53% Met, 61.25% Ile, 61.154% Leu, 61.82% Phe, 61.22% Lys, 59% Trp, respectively) present in the initial sample (iGAF t0) crossed the Caco-2 cells’ basolateral membrane, reaching the BL solution. Considering the initial iGAF t0 composition, the BL solution was particularly rich in BCAAs, (Val, Ile and Leu) and Phe.

### 3.2. Evaluation of Bioavailable iGAF Effects on the Activation of Akt-mTORC1-GSK3α/β: Key Targets of Anabolic and Metabolic Signaling Pathways

Considering the iGAF ability to across the differentiated Caco-2 cells, to investigate the bioavailable EAAs’ impact on myotubes’ protein synthesis and energetic metabolism, we developed a co-culture in which differentiated Caco-2 and differentiated C2C12 cells were employed. Briefly, intestinal Caco-2 cells were differentiated on the insert of the Transwell system, and C2C12 were simultaneously differentiated to myotubes. Caco-2 cells were moved on the differentiated C2C12 cells and treated with iGAF 10.7 mg/well for 2 h. In agreement with MTT results and the morphological observation that demonstrated the absence of negative effects on C2C12 cells’ viability (Figures S2 and S3, respectively), after 2 h, Caco-2 cells seeded on the Transwell insert in the AP chamber were removed, and the BL solution containing the EAAs that crossed the basolateral membrane was kept in contact overnight with myotubes. In muscle, Akt and mTORC1 have a strong positive correlation [22,23,24]. Indeed, Akt acts as a primary upstream activator, phosphorylating and turning on mTORC1, which then drives protein synthesis and cell growth (hypertrophy) and inhibits breakdown, making the Akt/mTOR pathway essential for muscle building and preventing atrophy, activated by factors like resistance exercise, amino acids, and growth factors. The results of the Western blot analysis conducted on C2C12 lysates show that the treatment with iGAF modulated the protein levels of Akt and p-Akt up to 110.1 ± 3.016 and 133.2 ± 8.678, compared to untreated control cells, respectively (Figure 2A,B), thus resulting in the augmentation of the p-Akt/Akt ratio up to 1.289 ± 0.05135% in C2C12 treated cells (Figure 2C). Moreover, results show that bioavailable iGAF increased the protein levels of mTOR and p-mTOR (Ser2448) up to 115.7 ± 16.72% and 144.8 ± 14.52%, respectively (Figure 2D,E). Indeed, the p-mTOR/mTOR ratio was 1.274 ± 0.08149% (Figure 2F), indicating that the bioavailable iGAF induced the activation of the mTORC1 complex, suggesting that Akt-mTOR anabolic signaling pathways were activated [25]. These results suggest that EAAs’ formulation positively stimulates protein synthesis and anabolic processes [26].

Hence, after observing that the bioavailable fraction of iGAF was able to significantly increase mTOR phosphorylation in differentiated C2C12 cells, we next examined its effects on additional key regulators of energy metabolism and anabolic signaling. Consistent with the role of Akt in promoting C2C12 hypertrophy through the inhibitory phosphorylation of its substrate, GSK3 [27], phosphorylation of GSK3 was also elevated, indicating inhibition of this kinase and the consequent release of its negative control on processes such as protein synthesis and glycogen accumulation [28]. Data of Western blot experiments conducted on differentiated C2C12 lysates demonstrated that treatment with iGAF did not induce a significant increase in protein levels for GSK3β (Figure 2G), while an increase in p-GSK3 protein levels of up to 173.4 ± 17.77% compared to untreated control cells was observed (Figure 2H). Overall, the p-GSK3/GSK3 ratio was increased to 1.538 ± 0.1040 (Figure 2I).

### 3.3. Effects of Bioavailable iGAF on AMPK Activation and Energy Metabolism

In parallel, our data demonstrated that iGAF did not induce an increase in AMPK protein levels (Figure 3A) but could significantly increase the p-AMPKα (Thr172) protein levels by up to 187.6 ± 36.95% (Figure 3B). Consequently, the p-AMPKα (Thr172)/AMPK ratio was significantly increased by up to 1.732 ± 0.1414% (Figure 3C), compared to control conditions, suggesting the involvement of an energy-sensing response that can be co-activated alongside anabolic signaling, leading to an adjustment of energy-supportive pathways, helping to sustain the metabolic demand associated with enhanced anabolic activity [29]. Overall, these results show that iGAF can modulate several interconnected metabolic nodes, contributing to a broader shift in cellular energy regulation in C2C12 cells.

### 3.4. iGAF Cell Lines Positively Modulate GLUT-4 and Functional Glucose Uptake in Myotubes

Western blot analyses performed on C2C12 lysates collected from the basolateral compartment of the Caco-2/C2C12 co-culture system showed that the bioavailable iGAF increased GLUT4 protein levels by 171.4 ± 31.49% compared to control cells (Figure 4A). From a functional point of view, this molecular effect was accompanied by a 200.6 ± 4.373% increase in extracellular environment glucose uptake, indicating an enhanced cellular capacity for glucose absorption and handling (Figure 4B). These findings are consistent with the observed improvement in phosphorylated Akt and AMPK levels, respectively, which are key components of the signaling cascade that ultimately promotes GLUT4 translocation to the plasma membrane and its functionality [30].

## 4. Discussion

Our study aimed to investigate iGAF’s bioavailability and the metabolic impact of its bioavailable fraction on differentiated C2C12 myotubes using a dedicated designed Caco-2/C2C12 co-culture system. At this scope, differentiated Caco-2 cells were employed since they are a well-known in vitro model to study bioactives’ bioavailability. Firstly, the results in Figure 1 showed that iGAF had a high degree of intestinal bioavailability, with more than 50% of each amino acid crossing the Caco-2 monolayer within 2 h. This finding is consistent with the known rapid intestinal transport of free-form essential amino acids and aligns with our previous observations showing preserved amino acid integrity and high bioaccessibility following simulated gastrointestinal digestion [13]. Notably, the BL fraction was particularly enriched in BCAAs and phenylalanine, reflecting both their relative abundance in the formulation and their efficient transport across enterocytes (Figure 1). These results confirm that iGAF provides a readily absorbable source of EAAs, supporting its suitability for conditions requiring rapid amino acid delivery, such as post-exercise recovery, metabolic stress or age-related anabolic resistance [31,32].

Then, the development of our innovative experimental approach, which couples the simulated gastrointestinal digestion with the co-culture system composed by the intestinal absorption model (Caco-2) and the skeletal muscle cell function model (C2C12), represents a key methodological strength of the present study. This combined in vitro strategy allows for the simultaneous evaluation of amino acids’ bioavailability and downstream biological activity at the target tissue level, thereby more closely resembling the physiological crosstalk that occurs in vivo. Differently from single-cell-type assays, the co-culture framework accounts for intestinal transport, metabolic transformation, and effective cellular exposure prior to assessing functional outcomes in muscle cells, offering a mechanistically informed platform for evaluating the in vitro bioactivity of nutritional ingredients and food supplements. Indeed, the dynamic exposure of C2C12 myotubes to the bioavailable iGAF fraction elicited a marked activation of mTORC1 signaling, as evidenced by the increased levels of mTOR and its phosphorylated form (Figure 2D,E). This observation aligns with the well-established role of EAAs, particularly leucine [33,34], in stimulating mTORC1 via Rag GTPase-mediated lysosomal translocation and underscores the capacity of the absorbed fraction to act as a direct anabolic stimulus. mTORC1 activation is positively correlated with the observed increase in the p-Akt/Akt ratio and enhanced phosphorylation of GSK3 (Figure 2C,H), a downstream effector of Akt whose inhibition promotes protein synthesis and glycogen accumulation [35]. Together, these findings indicate that the bioavailable iGAF activates the anabolic response at a cellular level, suggesting a synergistic contribution of multiple EAAs beyond leucine alone.

Interestingly, iGAF treatment also significantly increased the p-AMPK/AMPK ratio (Figure 3C). While AMPK is traditionally viewed as a negative regulator of mTOR, growing evidence shows that AMPK activation may co-occur with anabolic signaling in circumstances of increased energetic demand, serving to restore ATP levels and support biosynthetic processes [36]. In this context, AMPK activation may reflect a compensatory response to the enhanced metabolic requirements imposed by Akt/mTOR-driven anabolism or may arise from the intrinsic metabolic effects of specific EAAs. The simultaneous engagement of mTORC1, Akt, GSK3, and AMPK suggests that iGAF promotes a complex metabolic rewiring in myotubes, whereby anabolic stimulation is supported by enhanced energy-sensing and substrate-handling mechanisms.

Consistent with this interpretation, the bioavailable iGAF induced a significant increase in GLUT4 protein levels and significantly enhanced glucose uptake in C2C12 cells (Figure 4A,B). These results align with the activation of both Akt and AMPK signaling pathways, as both Akt and AMPK independently promote GLUT4 translocation to the plasma membrane [37,38]. The observed increase in glucose utilization may reflect an improved capacity of myotubes to meet the energetic demands associated with protein synthesis and metabolic activation. These results further integrate with our previous findings, demonstrating iGAF’s ability to inhibit DPP-IV activity, stimulate GLP-1 secretion and enhance incretin stability at the intestinal level. Taken together, the present and previous data suggest that iGAF exerts a multi-level action on metabolic regulation, combining intestinal effects on incretin physiology with direct peripheral actions on muscle energy metabolism.

## 5. Conclusions

Overall, the results provide novel insights into the mechanisms through which Gunaminoformula, an EAA blend, after in vitro digestion and bioavailability studies, may influence muscle metabolism at a cellular level. The high intestinal permeability of iGAF, combined with its ability to activate key anabolic signaling pathways and enhance glucose uptake in myotubes, suggests that it has potential applications in supporting in vitro muscle function. Lastly, while the co-culture system provides preliminary mechanistic insights, in vivo studies are needed to evaluate the EAAs’ blend effectiveness, particularly in conditions characterized by anabolic impairment or metabolic dysfunction. Future studies should aim to evaluate whether these in vitro effects translate into measurable physiological benefits in vivo.

## Data Availability

The original contributions presented in this study are included in the article/Supplementary Materials. Further inquiries can be directed to the corresponding author.

## Supplementary Materials

The following supporting information can be downloaded at https://www.mdpi.com/article/10.3390/nu18020323/s1, Figure S1: TEER Measurements in Differentiated Caco-2 Cells; Figure S2. Evaluation of iGAF’s impact on C2C12 cells viability; Figure S3. Morphological representation of differentiated C2C12 cells.

## Abbreviations

The following abbreviations are used in this manuscript:

Akt	Protein kinase B
ALA	Alanine
AMPK	AMP-activated protein kinase
ARG	Arginine
ASN	Asparagine
ASP	Aspartic acid
BCAAs	Branched-chain amino acids
BSA	Bovine Serum Albumin
C2C12	Murine skeletal myoblast cell line
Caco-2	Human epithelial colorectal adenocarcinoma cell line
CYS	Cysteine
DMEM	Dulbecco’s Modified Eagle Medium
DPP-IV	Dipeptidyl peptidase-IV
EAAs	Essential amino acids
FBS	Fetal Bovine Serum
GAF	Gunaminoformula
GLN	Glutamine
GLP-1	Glucagon-like peptide-1
GLU	Glutamic acid
GLUT4	Glucose transporter type 4
GLY	Glycine
GSK3	Glycogen synthase kinase 3
HBSS	Hank’s Balanced Salt Solution
HEPES	4-(2-hydroxyethyl)-1-piperazineethanesulfonic acid
HIS	Histidine
HRP	Horseradish peroxidase
iGAF	Gunaminoformula after INFOGEST digestion
ILE	Isoleucine
INFOGEST	Standard in vitro gastrointestinal digestion protocol
LEU	Leucine
LYS	Lysine
MES	2-(N-morpholino)ethanesulfonic acid
MET	Methionine
mTOR	Mechanistic target of rapamycin
mTORC1	Mechanistic target of rapamycin complex 1
MTT	(3-(4,5-dimethylthiazol-2-yl)-2,5-diphenyltetrazolium bromide)
NBDG	2-(N-(7-nitrobenz-2-oxa-1,3-diazol-4-yl)amino)-2-deoxyglucose
p-Akt	Phosphorylated Akt
p-AMPK	Phosphorylated AMPK
PBS	Phosphate Buffered Saline
PHE	Phenylalanine
PMSF	Phenylmethylsulfonyl fluoride
PRO	Proline
RIPA	Radioimmunoprecipitation assay buffer
SDS-PAGE	Sodium dodecyl sulfate polyacrylamide gel electrophoresis
SER	Serine
TEER	Transepithelial Electrical Resistance
THR	Threonine
TRP	Tryptophan
TYR	Tyrosine
VAL	Valine
WB	Western Blot

## Appendix A

**Table A1:** Gunaminoformula nutritional composition.

Nutritional Information	Per 100 g	Per 5 Tablets
Energy	1676 kJ/395 kcal	85 kJ/20 kcal
Fats	0.07 g	0 g
of which saturated fatty acids	0 g	0 g
Carbohydrates	0 g	0 g
of which sugars	0 g	0 g
Proteins	0 g	0 g
Salt	0.01 g	0 g
L-Leucine	19.70 g	1000 mg
L-Valine	15.76 g	800 mg
L-Isoleucine	14.78 g	750 mg
L-Lysine	13.79 g	700 mg
L-Phenylalanine	12.81 g	650 mg
L-Threonine	10.84 g	550 mg
L-Methionine	6.90 g	350 mg
L-Tryptophan	3.94 g	200 mg
