# Supplementary material for: A Molecular and Functional Investigation of the Anabolic Effect of an Essential Amino Acids’ Blend Which Is Active In Vitro in Supporting Muscle Function
Source: Nutrients. 2026 Jan 20;18(2):323. doi: 10.3390/nu18020323 (PMC12844987; doi:10.3390/nu18020323)
Supplement: Supplementary file 1 [file nutrients-18-00323-s001.zip › nutrients-4082946-Supplementary material.pdf]

Supplementary material

Figure S1. TEER Measurements in Differentiated Caco-2 Cells

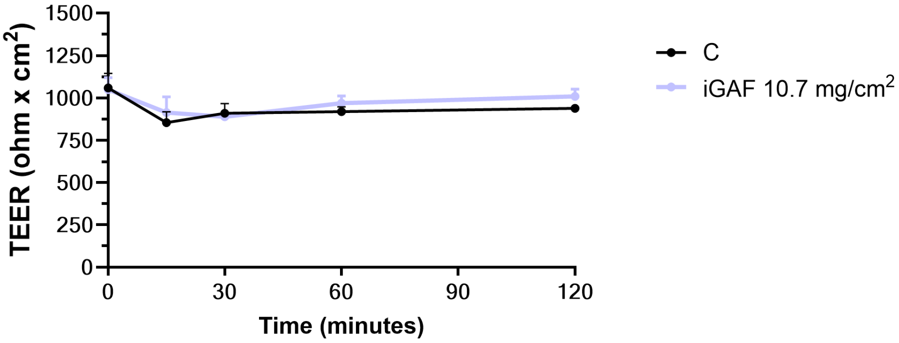

Figure S1. Transepithelial electrical resistance (TEER) measurements in differentiated Caco-2 monolayer. Black line refers to the untreated Caco-2 cells (C), lilac line to Caco-2 cells trated with iGAF (iGAF 10.7 mg/cm²). Data are the mean ± S.D. of three indipendent experiments performed in triplicate (biological replicates). C: untreated cells. iGAF: INFOGEST Gunaminoformula.

S2. Evaluation of iGAF's impact on C2C12 cells viability

Before proceeding with the experiments on the co-culture Caco-2/C2C12 cells, it was necessary to perform MTT assays to verify that iGAF did not affect C2C12 cells viability. The results (Figure S2) indicated that the sample was safe for differentiated C2C12 at a dose of 10 mg/cm² (corresponding to 3 mg in 0.32 cm² in a 96-well plate). These findings show that the sample directly tested on myotubes did not impair cells viability.

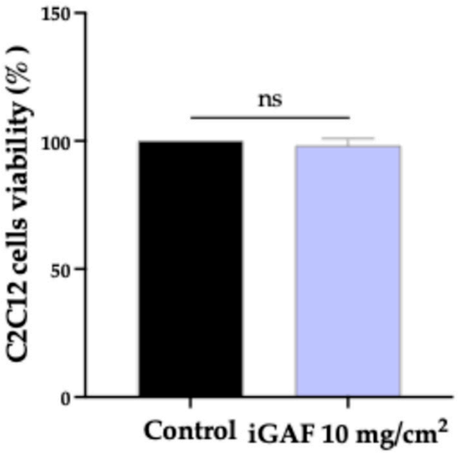

Figure S2. Evaluation of iGAF on differentiated C2C12 cells viability. Black line refers to control cells, lilac line refers to cells treated with iGAF 10 mg/cm^2. Data represent the mean ± s.d. of six determinations performed in triplicate. All the data sets have been analyzed by t-test. ns: not significant; Control: untreated cells; iGAF: GAF sample subjected to INFOGEST protocol. ns: not significant.

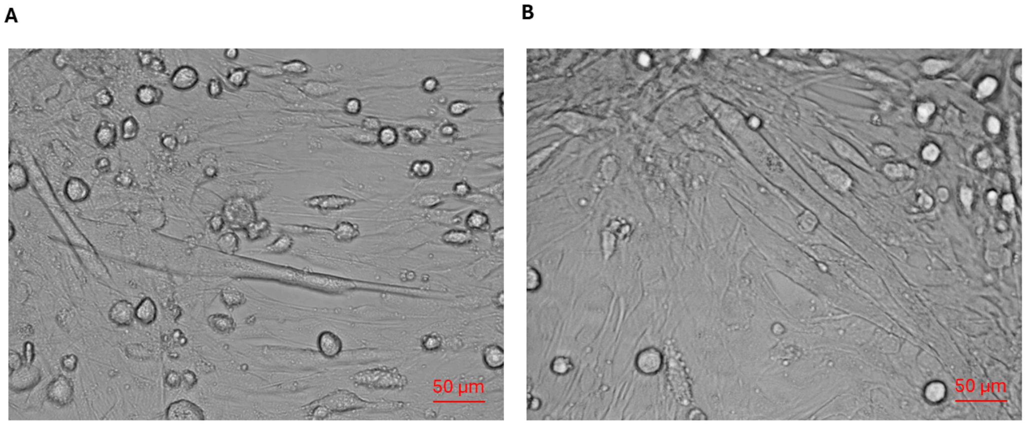

Figure S3. – Morphological representation of differentiated C2C12 cells. A) Microscopy image of untreated C2C12 cells showing elongated myotubes morphology; B) differentiated C2C12 cells after the treatment with iGAF 10 mg/cm^2. Both the pictures have been obtained at 20X magnification with Axio Vert.A1 microscope and ZEN Microscopy Software. Scale bar 50 µm.
